# Analysis of Clinical and Genetic Factors of Obesity and Psoriasis Concomitance—The Influence of Body Mass Composition, Prevalence of Mood Disorders, Environmental Factors and *FTO* Gene Polymorphisms (rs9939609, rs1558902)

**DOI:** 10.3390/biomedicines12030517

**Published:** 2024-02-25

**Authors:** Anna Czarnecka, Dorota Purzycka-Bohdan, Monika Zabłotna, Roman J. Nowicki, Krzysztof Rębała, Michał Bohdan, Marcin Gruchała, Alina Wilkowska, Aneta Szczerkowska-Dobosz

**Affiliations:** 1 Department of Dermatology, Venereology and Allergology, Medical University of Gdańsk, 80-210 Gdańsk, Poland; dorota.purzycka-bohdan@gumed.edu.pl (D.P.-B.); monika.zablotna@gumed.edu.pl (M.Z.); rnowicki@gumed.edu.pl (R.J.N.); aneta.szczerkowska-dobosz@gumed.edu.pl (A.S.-D.); 2 Department of Forensic Medicine, Medical University of Gdańsk, 80-210 Gdańsk, Poland; krzysztof.rebala@gumed.edu.pl; 3 1st Department of Cardiology, Medical University of Gdańsk, 80-210 Gdańsk, Poland; michal.bohdan@gumed.edu.pl (M.B.); marcin.gruchala@gumed.edu.pl (M.G.); 4 Department of Psychiatry, Medical University of Gdańsk, 80-210 Gdańsk, Poland; ali.wilkowska@gmail.com

**Keywords:** psoriasis, overweight, obesity, depression, BIA, *FTO* polymorphisms

## Abstract

This study aimed to comprehensively analyze the problem of overweight and obesity among psoriatic patients by investigating the influence of body mass composition, anhedonia and depression, environmental factors and *FTO* gene polymorphisms. Methods: The study enrolled 30 overweight or obese adult patients with chronic plaque psoriasis and 30 overweight or obese volunteers (northern Poland region, Caucasian population). Mood disorders, body mass composition by using bioelectrical impedance analysis (BIA) and *FTO* gene polymorphisms (rs9939609, rs1558902) by tetra-primer amplification refractory mutation system PCR (T-ARMS-PCR) were assessed. Results: Results revealed significantly higher visceral adipose tissue levels in psoriatic patients (5.23 ± 2.29 [L] vs. 3.41 ± 1.86 [L]), *p* = 0.001), especially among men, along with elevated rates of moderate and severe depression (26.67% vs. 6.67% and 13.33% vs. 3.33%, *p* = 0.048 respectively). Additionally, *FTO* gene polymorphisms correlated with waist–hip ratio differences in both groups. Conclusions: The study highlights the importance of evaluating body composition beyond body mass index, recognizing its influence on psoriasis and associated conditions like depression. The *FTO* gene may serve as a potential genetic link between psoriasis and obesity, warranting further research for validation. Adiposity emerges as a key and modifiable risk factor, underscoring the clinical implications of body composition complexities in psoriasis management.

## 1. Introduction

Psoriasis, a prevalent immune-mediated inflammatory skin disease characterized by a chronic course and multifactorial etiology [1], has been extensively studied in the scientific literature. Existing research has firmly established the impact of immunological factors, including the cascade production of proinflammatory cytokines such as tumor necrosis factor alpha (TNF-α), interferon alpha (INF-α), interleukin 6 (IL-6), activation of Th1 and Th17 lymphocytes and key mediators like interleukin 17 (IL-17), interleukin 12 (IL-12) and interleukin 23 (IL-23) [2,3]. Environmental factors (encompassing emotional stress, smoking habits, lifestyle, diet, physical activity and infections), as well as genetic background (notably the HLA-Cw6*06 allele) are also well-documented contributors to its pathology [4,5].

Currently, chronic plaque psoriasis is considered as a systemic disorder primarily attributed to persistent systemic inflammation [6]. This inflammatory process is recognized as a fundamental element contributing to psoriasis comorbidities, such as obesity, hypertension, diabetes, metabolic syndrome, cardiovascular disease (CVD) and depression [7,8]. Compelling evidence suggests that obesity plays a pivotal role as a triggering factor in the development of psoriasis comorbidities [9]. This association may arise not only from shared inflammatory pathways, but also from a common genetic background [10,11].

Currently, the *FTO* gene, located on chromosome 16, is regarded as a key player in the genetics of obesity, with significant impact revealed in genome-wide association studies [12]. Its association with body mass index (BMI), hip circumference and body weight has been established [13]. The *FTO* gene codes for a 2-oxoglutarate-dependent nucleic acid demethylase, a protein involved in regulating food intake and body energy expenditure, thereby influencing body fat mass control. Notably, the highest FTO mRNA expression is found in hypothalamic nuclei, crucial for regulating energy homeostasis [14].

The potential shared genetic background between obesity and psoriasis remains elusive, with limited studies exploring the role of the *FTO* gene in the pathogenesis of obesity among psoriatic patients, including the Polish psoriasis population [15,16,17].

Beyond genetic factors, the interplay between obesity and psoriasis may arise from dysregulated proinflammatory and metabolic molecular pathways, influenced by body mass composition, mood disorders or environmental factors such as nutritional habits, cigarette smoking and physical activity [18]. Mood disorders and depression, in particular, are recognized as significant contributors to increased cardiovascular risk, with epidemiological studies indicating a higher prevalence among psoriasis patients compared to the general population [7,19]. Furthermore, detailed analysis of body mass composition using the bioelectrical impedance method (BIA) is the latest trend in modern research and allows, among others, precise measurement of the amount of visceral and abdominal fat tissue. It is postulated that the previously used indicators of excess body weight, such as BMI, are insufficient to precisely define the metabolic management of patients [20].

In this study, we aimed to comprehensively analyze the problem of overweight and obesity among psoriatic patients by investigating the influence of: (a) body mass composition as an individual trigger, (b) anhedonia and depression as psychiatric triggers, (c) diet, smoking and physical activity as environmental triggers, and (d) *FTO* gene polymorphisms as genetic triggers.

## 2. Materials and Methods

### 2.1. Study Group

A cross-sectional study was carried out between 2021 and 2023 at the Department of Dermatology, Venereology and Allergology of the Medical University of Gdańsk and at the Dermatological Outpatient Clinic in cooperation with the 1st Department of Cardiology of the Medical University of Gdańsk and the Division of Psychiatry of the Medical University of Gdańsk. The patient and control groups enrolled 30 unrelated adult patients with clinical and/or histological diagnosis of psoriasis vulgaris who were overweight or obese and 30 overweight or obese unrelated adult volunteers, respectively (northern Poland region, Europe, Caucasian population). The mean age of psoriatic patients was 41.07 ± 12.58 years (age range 18.0–64.0 years). Among those individuals, there were 4 (13.33%) women (mean age 40.75 ± 6.29 years, age range 36.0–50.0 years), and 26 (86.67%) men (mean age 41.12 ± 13.37 years, age range 18.0–64.0 years, women vs. men: *p* = 0.95). The mean age of control population was 42.33 ± 18.25 years (age range 20.0–81.0 years), out of which 10 (33.33%) were women (mean age 39.30 ± 16.06 years, age range 21.0–61.0 years) and 20 (66.66%) were men (mean age 43.85 ± 19.46 years, age range 20.0–81.0 years, women vs. men: *p* = 0.58). The inclusion criteria for the patients’ group involved: age ≥18 y/o, chronic plaque psoriasis diagnosis, overweight or obesity (defined as BMI ≥ 25 kg/m^2^), minimal 6-month withdrawal period from systemic psoriatic treatment, no other systemic concomitant diseases (including neoplastic, myeloid and metabolic diseases, such as hypertension, diabetes, cardiovascular disorder or bone marrow transplantation). The inclusion criteria for the control group involved: age ≥18 y/o, no chronic plaque psoriasis diagnosis or positive family history, overweight or obesity (defined as BMI ≥ 25 kg/m^2^), no other systemic concomitant diseases (including neoplastic, myeloid and metabolic diseases, such as hypertension, diabetes, cardiovascular disorder or bone marrow transplantation).

The research protocol was authorized by the Independent Bioethics Committee for Scientific Research (NKBBN/2/2021 and NKBBN/953/2021). All subjects provided written, informed consent prior to their partaking in the study.

### 2.2. Data Collection, Anthropometric and Laboratory Measurements

Demographic and clinical data were collected from each participant of the study, including the influence of environmental factors (dietary habits, cigarette smoking status, level of physical activity), impact of psoriasis on health-related life quality (defined by DLQI—Dermatological Life Quality Index) [21] and the presence of mood disorders and anhedonia (using certified questionnaires: PHQ-9—Patient Health Questionnaire-9 [22], SHAPS—Snaith-Hamilton Pleasure Scale [23]). Medical examination with assessment of the nutritional status (weight, height, waist and hip circumference, WHR—waist-to-hip ratio, BMI and RFM—relative fat mass index) and the severity of psoriasis (PASI—Psoriasis Area Severity Index [24], BSA—Body Surface Area) or the presence of skin lesions (control group) was performed. The study involved assessment of the body mass composition using bioelectrical impedance analysis (BIA) in accordance with the manufacturer’s procedures and algorithms (certified medical scale SECA mBCA 514/515, SECA Gmbh & Co. Kg, Hamburg, Germany). From each subject, peripheral blood sample was collected for lipid profile (total cholesterol, triglyceride (TG), low-density lipoprotein cholesterol (LDL), high-density lipoprotein cholesterol (HDL)) and genetic analysis. The definition criteria applied in the study are summarized in Table S1 of Supplementary Materials and follow World Health Organization (WHO) standards. 

### 2.3. Identification of FTO Gene Polymorphisms (SNP rs9939609 A/T and SNP rs1558902 A/T)

Genomic DNA was extracted from whole-blood specimens using Blood DNA Prep Plus, following the manufacturer’s protocol (A&A Biotechnology, Gdynia, Poland). *FTO* gene polymorphisms were assessed using the tetra-primer amplification refractory mutation system PCR (T-ARMS-PCR) technique, employing custom-designed oligonucleotide sequences for *FTO* rs1558902 (Supplementary Materials Table S2) and previously described primers for *FTO* rs9939609 [25]. The PCR conditions were as follows: initial denaturation for 5 min at 94 °C; 34 cycles of 50 s at 94 °C, annealing step for 60 s at 61 °C, and 60 s at 72 °C; and final elongation at 72 °C for 5 min. The PCR products were separated in a 2% agarose gel.

### 2.4. Statistical Analysis

For statistical analysis and data presentation, Statistica v. 12.0. (StatSoft, Inc., Tulsa, OK, USA, 2015) and Microsoft Office Excel (Microsoft Corporation, Redmond, WA, USA, 2018, version 16.16.27) were used. χ^2^ analysis was employed to compare the actual number of genotypes with the expected values for a population in Hardy–Weinberg equilibrium, as well as to assess the significance of variations in observed genotypes and alleles among the study groups [26]. Qualitative feature analyses were conducted using the χ^2^ test with Pearson’s method. Independent variables that met the assumptions for parametric tests were analyzed using Student’s *t*-test. Independent variables that did not meet the assumptions for parametric tests were analyzed using non-parametric tests, including the Mann–Whitney U test or the Kruskal–Wallis test. The relationship between quantitative variables was examined using the Spearman’s rank correlation. A *p*-value <0.05 was considered indicative of statistical significance. 

## 3. Results

### 3.1. Study Group Characteristics

Psoriasis group characteristics are provided in Table 1. Among the psoriatic patients, 11 (36.67%) reported other comorbid diseases (summary presented in Supplementary Material Figure S1). Current or previous psoriasis treatment methods were evaluated (all patients (100.00%) topical corticosteroids; 17 (56.57%) methotrexate; 11 (36.67%) cyclosporine A; 10 (33.33%) phototherapy; 7 (23.33%) acitretin; 6 (20.00%) biological therapies). Eight (26.67%) control subjects reported other comorbid diseases (summary presented in Supplementary Materials Figure S1). 

### 3.2. Anthropometric Measurements and Bioelectrical Impedance Analysis

Based on BMI results, in the psoriasis group, 12 (40.0%) subjects were overweight and 18 (60.0%) were obese. Among overweight subjects, mean BMI was 27.77 ± 1.41 (range 26.03–29.92), with 10 (83.33%) men and 2 (16.67%) women (*p* = 0.66). Among obese subjects, mean BMI was 34.87 ± 4.10 (range 29.99–42.34), with 16 (88.89%) men and 2 (11.11%) women (*p* = 0.66).

In the control group, 16 (53.33%) subjects were overweight and 14 (46.67%) were obese based on BMI. Among overweight subjects, mean BMI was 27.35 ± 1.48 (range 25.1–29.8), with 11 (63.75%) men and 5 (31.25%) women (*p* = 0.8). Among obese subjects, mean BMI was 32.92 ± 9.66 (range 31.7–46.02), with 9 (64.29%) men and 5 (35.71%) women (*p* = 0.8).

Evaluation of anthropometric measurements among the study group is presented in Table 2. Notable discrepancies were observed in waist circumference and WHR of men between the psoriatic and control groups (113.54 cm vs. 104.03 cm, *p* = 0.02; 1.01 vs. 0.92, *p* = 0.001, respectively) and in the relative fat mass index among men and women in the psoriasis and control groups (31.68% vs. 42.29%, *p* = 0.003; 28.54% vs. 42.48%, *p* = 0.00002, respectively).

**Figure 1:**
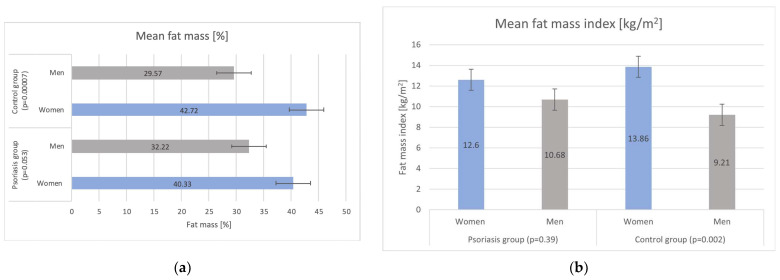
Analysis of (**a**) mean fat mass (FM) and (**b**) mean fat mass index (FMI) in the study groups.

Bioelectrical impedance analysis showed tendency in women for greater fat mass (FM) and fat mass index (FMI) in comparison to men, regardless of psoriasis disease (data presented in Figure 1). There were no statistically significant differences among men and women in the context of mean FM and FMI between the psoriasis and the control group (difference between mean FM and mean FMI in psoriasis and controls (comparison of mean FM and mean FMI in psoriasis and controls: *p* = 0.66 and *p* = 0.79, respectively); however, psoriasis men had slightly higher FM and FMI than control men.

**Figure 2:**
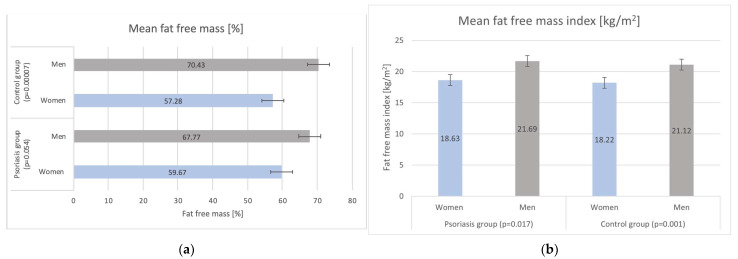
Analysis of (**a**) mean fat free mass (FFM) and (**b**) mean fat free mass index (FFMI) in the study groups.

Furthermore, no statistically relevant discrepancies in mean fat free mass (FFM) were reported between psoriatic and control groups (*p* = 0.66); however, a higher mean fat free mass index (FFMI) was observed for psoriatic patients than control subjects (21.37 ± 2.15 vs. 20.15 ± 3.31, *p* = 0.04). In general, men had increased FFM and FFMI in comparison to women (Figure 2).

Total body water (TBW), extracellular body water (ECW) and the ECW/TBW ratio did not deviate from standardized norms for the whole study group (data available in Table 3). In general, TBW [L] and ECW [L] was higher in psoriasis patients than in controls (51.1 ± 7.51 [L] vs. 46.16 ± 8.52 [L], *p* = 0.017; 21.57 ± 2.99 [L] vs. 19.69 ± 3.48 [L], *p* = 0.03 respectively). In the control group, both TBW [L, %] and ECW [L, %] values were significantly higher in men than women. In psoriasis group, only ECW [L] was increased in men in comparison to women. However, there were no statistically significant differences in the ECW/TBW ratio between psoriasis and control groups (42.12 ± 1.41 [%] vs. 42.85 ± 2.52 [%], *p* = 0.18). 

**Figure 3:**
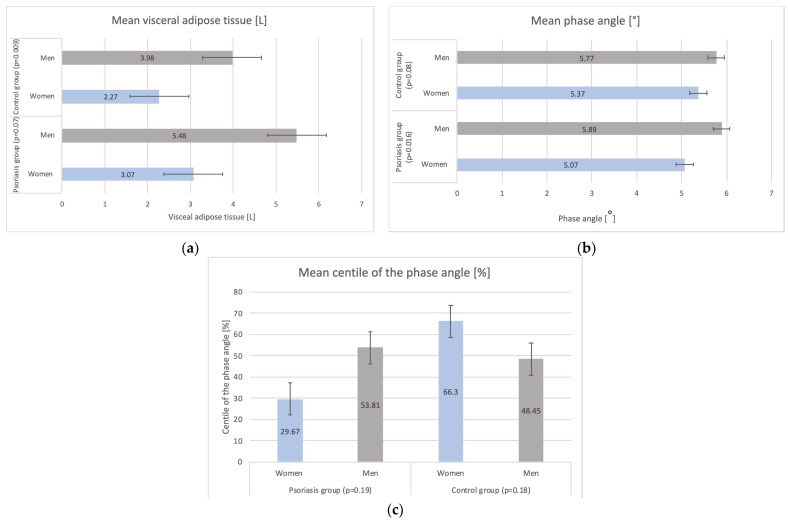
Analysis of the mean (**a**) visceral adipose tissue (VAT), (**b**) phase angle (PhA) and (**c**) centile of the phase angle (cPhA) in the study groups.

In general, the psoriasis population had a significantly higher mean visceral adipose tissue level (VAT) than the control population (5.23 ± 2.29 [L] vs. 3.41 ± 1.86 [L]), *p* = 0.001) (Figure 3), especially among men (5.48 vs. 3.98 [L], *p* = 0.02) (Figure 3). Moreover, there was no significant difference in mean phase angle (PhA) between the control population and psoriatic patients (5.63 vs. 5.81, *p* = 0.38), whereas psoriatic women tend to have a smaller phase angle than control women (5.37 vs. 5.07, *p* = 0.18). Mean centile of the phase angle (cPhA) analysis did not provide statistically significant results. 

Correlation analyses were performed, investigating the relationship between body mass components (FM, FMI, VAT, PhA and cPhA) and severity of psoriasis (PASI and BSA). Statistically relevant results of positive correlations were established for: women between PASI and FM (*n* = 3, Spearman R = 0.9993, *p* = 0.023); women between PASI and cPhA (*n* = 3, Spearman R = 0.9994, *p* = 0.023); and psoriatic patients in total between BSA and VAT (*n* = 29, Spearman R = 0.395186, *p* = 0.03).

### 3.3. Anhedonia and Depressive Symptom Prevalence

**Figure 4:**
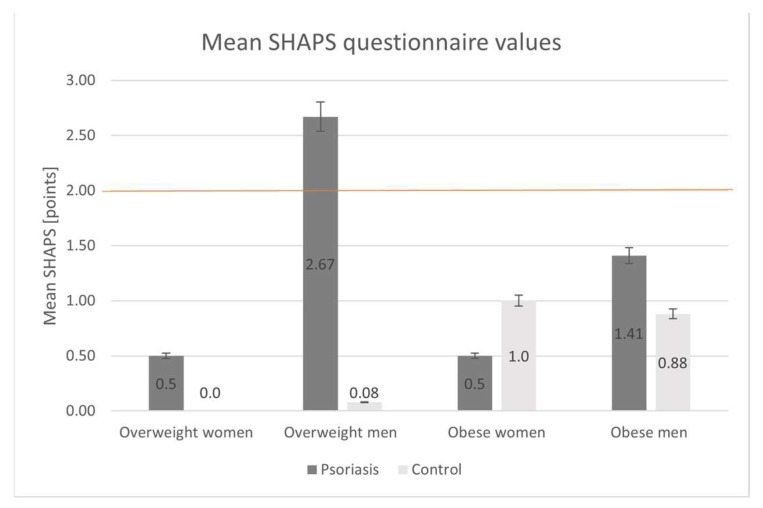
Analysis of Snaith-Hamilton Pleasure Scale (SHAPS) questionnaire results in the study groups (psoriatic group vs. control group: *p* = 0.02). Orange line represents the threshold of clinically relevant anhedonia (SHAPS >2 points).

Mean SHAPS questionnaire results are presented in Figure 4. From the whole study group, only 7 (11.67%) overweight individuals had SHAPS values indicating clinically relevant anhedonia (defined as SHAPS >2 points). Among them, 6 men (23.08%) had psoriasis and only 1 man (0.05%) was from the control group.

**Figure 5:**
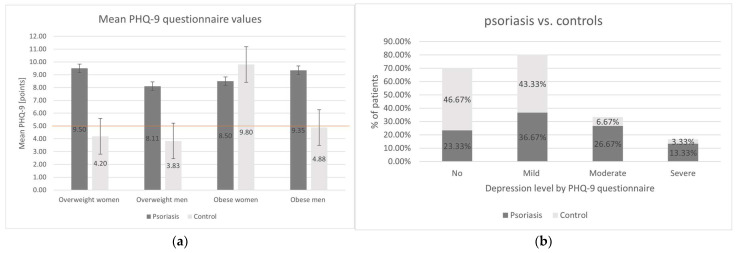

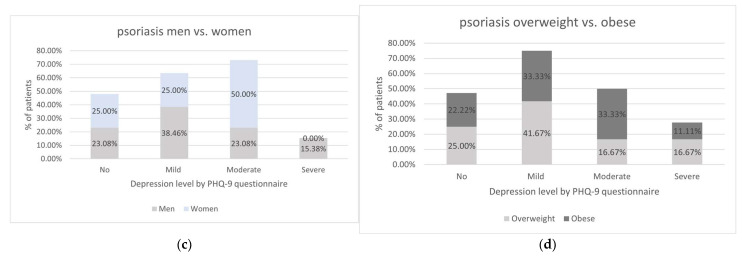
Analysis of Patient Health Questionnaire-9 (PHQ-9) questionnaire results in the study groups. (**a**) Mean PHQ-9 in the study group (psoriatic group vs. control group: *p* = 0.006). Orange line represents the threshold of mild depression (PHQ-9 ≥ 5 points). (**b**) Depression severity in psoriatic group vs. control group (*p* = 0.048). (**c**) Depression severity in psoriatic group men vs. women (*p* = 0.63). (**d**) Depression severity in psoriatic group overweight vs. obese individuals (*p* = 0.78).

In general, the mean PHQ-9 value was significantly higher in psoriatic patients than in the control group (8.93 ± 5.91 points vs. 5.17 ± 3.71 points, *p* = 0.006). In the control group, mean PHQ-9 exceeded a threshold of mild depression (PHQ-9 ≥5 points) only among obese women, whereas among psoriatic patients, it surpassed the threshold irrespective of sex and adipose tissue overabundance level (Figure 5). Furthermore, 76.67% of psoriatic patients had PHQ-9 indicating mood disorders, with mild depression (36.67%) being the most frequent. Also, moderate and severe depression prevalence was considerably higher among psoriatic patients than in the control group (26.67% vs. 6.67% and 13.33% vs. 3.33%, *p* = 0.048 respectively). Among psoriatic patients, men were predisposed to suffer from more severe symptoms than women (15.38% vs. 0.0%, *p* = 0.63), and obese psoriatic patients more than overweight ones (16.67% vs. 11.11%, *p* = 0.78), but the observed differences were not statistically significant.

### 3.4. Evaluation of Environmental Factors: Diet and Nutrition, Cigarette Smoking and Physical Activity

**Figure 6:**
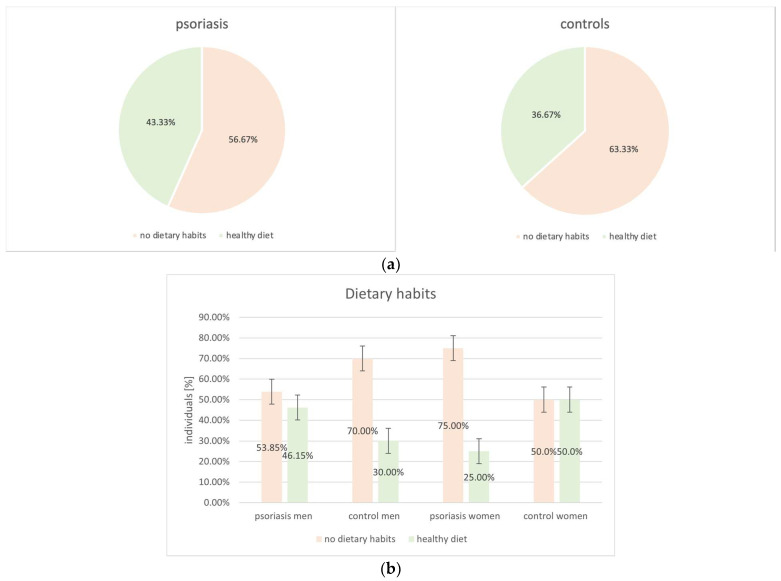
Analysis of dietary habits among the study groups (psoriatic group vs. control group: *p* = 0.6). (**a**) Psoriatic group vs. control group. (**b**) Men vs. women in regards to the study groups.

The majority of subjects, both psoriatic patients and controls, declared no healthy dietary habits (56.67% vs. 63.33%, *p* = 0.6) (Figure 6).

**Figure 7:**
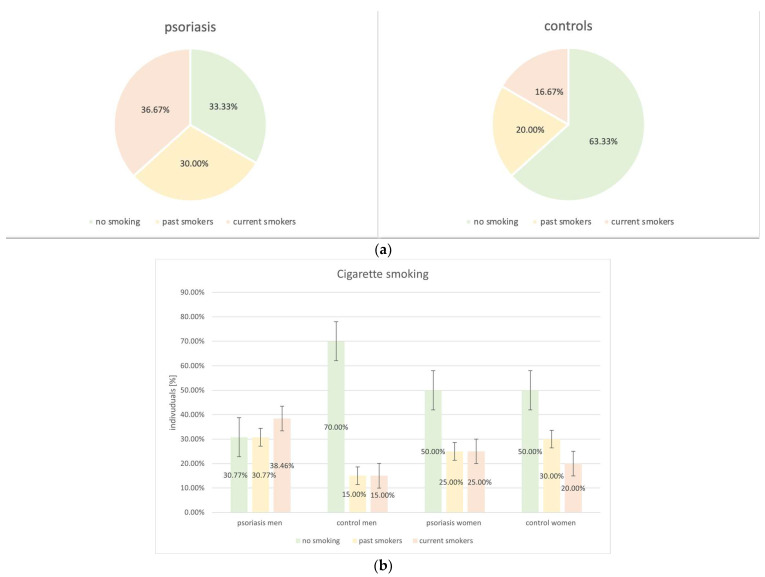
Analysis of cigarette smoking in the study groups (psoriatic group vs. control group: *p* = 0.06). (**a**) Psoriatic group vs. control group. (**b**) Men vs. women in regards to the study groups.

Among psoriatic patients, there was higher prevalence of past and current cigarette smokers in comparison to the control group (30.0% vs. 20.0% and 36.67% and 16.67%, *p* = 0.06 respectively). Furthermore, the cohort of psoriatic men had the greatest number of current smokers (38.46%) (Figure 7).

**Figure 8:**
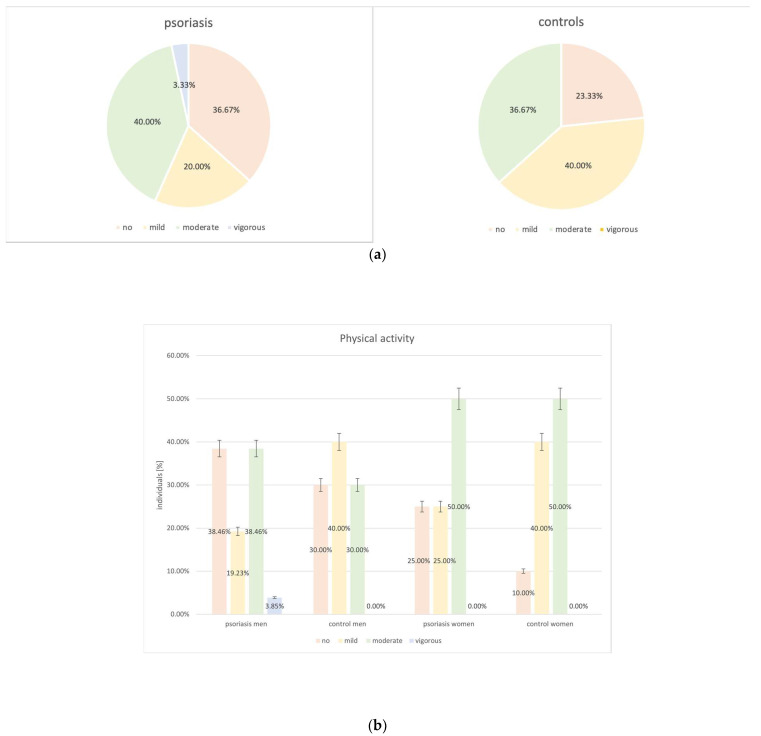
Analysis of physical activity levels among the study groups (psoriatic group vs. control group: *p* = 0.27). (**a**) Psoriatic group vs. control group. (**b**) Men vs. women in regards to the study groups.

Psoriatic patients declared to engage in less physical activity than the control group (63.33% vs. 76.67%, *p* = 0.27) (Figure 8). Control women were the most physically active group (90.0% declared mild or moderate physical activity), and the lowest level of physical activity was observed among psoriatic men (38.46% stated no physical activity).

### 3.5. Analysis of FTO Gene Polymorphism (rs9939609, rs1558902) Influence on Overweight, Obesity, Anhedonia and Mood Disorders and Disease Severity among Psoriatic Patients

The AA genotype of the *FTO* gene was the most prevalent in the studied population for both rs9939609 and rs1558902 polymorphisms, whereas the TT genotype was the least frequent. Table 4 and Table 5 summarize the association between the clinical parameters of overweight, obesity, depression and psoriasis disease severity for rs9939609 and rs1558902 polymorphisms of the *FTO* gene. 

#### 3.5.1. rs9939609 Polymorphism of the *FTO* Gene

In the psoriasis group, the TT genotype had greater mean BMI than the control population (30.17 ± 2.08 vs. 27.17 ± 1.88, *p* = 0.03). Although mean WC, FM, FMI and VAT value differences among the genotypes did not reach the statistical significance threshold, they were notably higher among AA and AT genotypes compared to the TT genotype. However, mean WHR values were greater for the AA genotype compared to AT + TT genotypes in the psoriatic group (1.02 ± 0.11 vs. 0.92 ± 0.09 and 0.98 ± 0.05, *p* = 0.06) and the AA genotype in the control group (1.02 ± 0.11 vs. 0.87 ± 0.05, *p* = 0.047). Higher mean RFM was associated with the AT genotype and lower mean RFM with the TT genotype (AT vs. AA + TT: *p* = 0.02; TT vs. AA + AT: *p* = 0.03). Also, greater prevalence of the AA genotype was found among patients with moderate and severe psoriasis (PASI, BSA and DLQI >10 points), but no statistical significance was observed. Mean PhA and SHAPS values were the highest among the TT homozygotes (5.98 ± 0.56, *p* = 0.04 and 4.17 ± 3.54, *p* = 0.006, respectively). Furthermore, the TT genotype was associated with greater prevalence of SHAPS > 2 score (TT vs. AA + AT: *p* = 0.04) among psoriatic patients and also when compared to the control population (*p* = 0.04). Mean PHQ-9 score for the TT genotype was greater among psoriatic patients than in the control population (14.33 ± 7.84 vs. 3.17 ± 2.04, *p* = 0.005), as was the prevalence of patients with PHQ-9 score > 4 (*p* = 0.03). 

In the control group, mean BMI and FMI values among the AA homozygotes were increased in comparison to TT individuals (31.10 ± 5.51 vs. 27.17 ± 1.88, *p* = 0.01 and 11.32 ± 3.88 vs. 7.97 ± 1.43, *p* = 0.02). Reversely, the mean WHR ratio was higher for TT homozygotes than AA homozygotes (0.96 ± 0.09 vs. 0.87 ± 0.05, *p* = 0.03; TT vs. AA + AT, *p* = 0.02). Although mean RFM and FM value differences among the genotypes did not meet the statistical significance threshold, they were notably higher for AA and AT genotypes than for TT genotypes. 

#### 3.5.2. rs1558902 Polymorphism of the *FTO* Gene

In the psoriasis group, mean WHR values were higher for AA and AT genotypes in comparison to the control group (0.99 ± 0.10 vs. 0.91 ± 0.06, *p* = 0.05; 1.02 ± 0.15 vs. 0.87 ± 0.07, *p* = 0.02, respectively). Among the AT heterozygotes, higher mean WC and VAT values were observed among the psoriatic population than in the control group (115.00 ± 11.63 vs. 99.82 ± 9.61, *p* = 0.03; 5.63 ± 2.31 vs. 3.06 ± 1.47 *p* = 0.03). All psoriatic patients with the AA genotype had BSA > 10% (*p* = 0.02). Furthermore, TT homozygotes had significantly lower mean BSA score and BSA > 10% compared to other genotypes (mean BSA TT vs. AA + AT: *p* = 0.006; BSA > 10% AA vs. AT + TT: *p* = 0.03; TT vs. AT + AA: *p* = 0.009). A notable association was observed for mean SHAPS values, with TT homozygotes having the highest score among all genotypes in the psoriatic patients (TT vs. AA + AT: *p* = 0.004) and when compared to the control group (*p* = 0.04). A greater number of AT genotype psoriatic patients reached PHQ-9 > 4 values in comparison to the control population (*p* = 0.045).

No significant associations were detected in the control group. 

## 4. Discussion

The escalating problem of overweight and obesity poses a significant challenge to global populations, with anticipated prevalence rates expected to rise to 20% for obesity and 38% for overweight individuals by the year 2030 [27]. Addressing the issue of overweight and obesity among patients with psoriasis emerges as a critical healthcare concern. The clinical implications of obesity in the context of psoriasis are linked to such factors as increased severity and early onset, reduced quality of life, compromised efficacy of psoriasis treatments and increased risk of morbidity, particularly through a shortened lifespan attributed to cardiovascular events [28,29,30]. The main findings of our study revealed significantly higher visceral adipose tissue levels in psoriatic patients (5.23 ± 2.29 [L] vs. 3.41 ± 1.86 [L]), *p* = 0.001), especially among men, along with elevated rates of moderate and severe depression (26.67% vs. 6.67% and 13.33% vs. 3.33%, *p* = 0.048 respectively). Additionally, *FTO* gene polymorphisms correlated with waist–hip ratio differences in both groups.

The composition of body mass represents a pivotal modifiable risk factor for obesity and exhibits noteworthy variations in the individuals with psoriasis. Specifically among men, those with psoriasis displayed elevated values of waist circumference (WC), waist-to-hip ratio (WHR) and relative fat mass (RFM) compared to the control population, indicating a pronounced inclination toward central obesity. Extensive observations from the Nurses’ Health Study have underscored the association between increased waist circumference and waist-to-hip ratio with an elevated risk of psoriasis in adults [31]. Moreover, Paller et al. confirmed this association in the pediatric population [32]. 

Notably, RFM values demonstrated consistency with fat mass outcomes across the entire study cohort, suggesting its potential as a more specific tool for diagnosing adiposity excess compared to the conventional BMI index. Its practical utility is underscored by its cost-effectiveness, speed of application and ease of use in routine clinical practice. Galluzzo et al. presented evidence supporting the superiority of bioelectrical impedance analysis over BMI in determining adipose tissue levels in psoriatic patients, emphasizing that individuals with normal-range BMI could still exhibit elevated adipose tissue levels [33].

While we did not observe significant differences in fat mass (FM) between psoriatic patients and controls, there was a notable increase in visceral adipose tissue (VAT) among the psoriatic population, particularly in the male group. The co-existent elevation of waist circumference (WC) and waist-to-hip ratio (WHR) may suggest a predisposition of psoriatic men to an accumulation of visceral adipose tissue, linked with augmentation of chronic inflammatory state [34] and an elevated risk of cardiovascular and metabolic mortality and morbidity [35]. The HUNT Study in Norway also reported elevated mean levels of total body fat and visceral fat among individuals with psoriasis compared to the general population. Moreover, an association was suggested between the presence of the HLA-C*06:02 allele and lower levels of visceral fat [36]. This hypothesis was further supported by CT- and MRI-derived body composition techniques, both revealing increased visceral fat area, as well as visceral and abdominal fat tissue in psoriatic individuals compared to healthy subjects [37,38].

Although our study demonstrated a positive correlation between increased adiposity and psoriasis severity (VAT and BSA% in the psoriasis group, FM and PASI among psoriatic women), a systematic review by Blake et al. presented conflicting results and called for further elucidation of the causal link between body composition parameters and PASI [20]. Additionally, Galluzzo et al. found adipose tissue to be positively correlated only with the age and the disease onset of psoriatic patients, rather than with disease severity [33].

Additionally, the mean phase angle was observed to be lower in the control population compared to psoriatic patients. In contrast, Barrea et al. reported a smaller phase angle among psoriatic patients and higher prevalence of metabolic syndrome when compared to controls [39]. However, recent research that assessed the use of phase angle as a screening tool concluded that its interpretation is highly variable due to the contribution of multiple factors, such as fat, fat-free and muscle mass, or hydration status. Consequently, phase angle values among overweight and obese individuals may exhibit considerable variation, with lower values commonly observed compared to healthy gender-matched adults, as demonstrated in our study. Furthermore, the reduced phase angle among overweight and obese psoriatic patients may suggest a reduction in fat-free mass and an increased visceral fat mass [40].

Our research shows that depressive symptoms are more common in individuals with psoriasis, affecting 76.67% of patients according to the mean score of 8.93 (± 5.91 points) on the PHQ-9 questionnaire, which is notably higher when compared to the general population, especially for those experiencing moderate to severe symptoms (26.67% and 13.33%, respectively). These findings align with published epidemiological research conducted in Poland and worldwide, highlighting that mood disorders, particularly depression, are more prevalent in individuals with psoriasis than in the general population, estimated to be around 60% [41,42]. A nationwide Danish cohort study with a large population reported incidence rates for depression of 20.0 (95% CI 19.9–20.0), 23.9 (23.1–24.7) and 31.6 (29.5–33.8) for the reference population and mild and severe psoriasis, respectively. The study further concluded that comorbidities primarily contribute to the risk of new-onset depression in psoriasis, except for younger individuals with severe psoriasis, where psoriasis itself may be a risk factor [43]. Also, a large population-based study among US citizens reported a significantly higher PHQ-9 score among psoriatic patients compared to the general population (4.54 vs. 3.22) [44].

Moreover, the findings suggest that psoriatic men may exhibit a higher susceptibility to mood disorders compared to psoriatic women (15.38% vs. 0.0%, *p* = 0.63), particularly in the context of overweight or obesity. Consequently, body weight emerges as a significant cofactor influencing the prevalence of mood disorders among psoriatic patients, in addition to the severity of skin lesions. Interestingly, contrary to our results, a recent meta-analysis proposed a greater predisposition of female sex among psoriatic patients (OR = 1.62, 95% CI 1.09–2.40) [45]. A nationwide population-based cross-sectional study in Taiwan identified female sex and major comorbid diseases, including cardiovascular disease, as major risk factors associated with depression among psoriatic patients [46]. A systematic review by Pavlova et al. highlighted a positive association between high BMI and increased reports of depression and anxiety among patients with psoriasis [47]. Lada et al. observed an association between depression and increased neutrophil levels, indicating systemic inflammation, particularly among psoriatic women. This association could result from metabolic changes and visceral adiposity, reinforcing obesity as a significant confounder of depression among psoriatic patients [48]. The interplay of systemic inflammation is postulated to play a pivotal role in the co-occurrence of depression and psoriasis, a relationship further exacerbated by immune dysregulation provoked by obesity [49]. Currently, a neuroinflammatory process mediated by the overexpression of IL-17 is suggested to play a central role in the immune cycle of depression, obesity and psoriasis [50].

We observed unfavorable lifestyle habits among psoriatic patients, with more pronounced prevalence among men. This included poor dietary habits (56.67% of psoriatic patients), high incidence of cigarette smoking (66.67% past and current smokers within the psoriasis population), and a lower level of physical activity (36.67% reporting no physical activity at all). This is particularly concerning given the heightened tendency of psoriatic men toward abdominal adiposity and mood disorders. These findings align with our prior research conducted on a large-scale Polish population, both among individuals with psoriasis and the general population [41]. Hence, there is a crucial need to advocate for healthy lifestyle choices and prioritize patient education initiatives among individuals with psoriasis. Recent findings indicate the significance of incorporating antioxidant-rich foods into psoriatic patients’ diet and considering antioxidants as a potential therapeutic approach for managing inflammatory skin conditions [51]. Oxidative stress is believed to be a crucial factor in the inflammatory cascade of the pathological mechanism of psoriasis. Bakić et al. highlighted the advantages of a multimarker approach, incorporating factors related to metabolism (such as glucose and triglicerydes) and renal function (such as creatinine and urea), which may serve as predictors of psoriasis severity more effectively than individual examined biomarkers [52].

Genetic markers have the potential to predict comorbidities and treatment efficacy, thereby improving treatment outcomes. In our study, we aimed to explore whether two *FTO* gene polymorphisms, rs9939609 and rs1558902, could serve as potential genetic links between psoriasis and obesity. Both of these single nucleotide polymorphisms (SNPs) have been associated with obesity and BMI in both worldwide and Polish populations [53,54], although the underlying mechanism among psoriatic patients remains unknown. The frequency of alleles in the studied populations for both polymorphisms was comparable, with the A allele being the most prevalent. Consequently, it appears that psoriatic patients do not exhibit an increased frequency of risk alleles for both polymorphisms of the *FTO* gene. This hypothesis requires further investigation on a larger population. However, Coto-Segura et al. also reported a comparable prevalence of the risk allele of *FTO rs9930506* polymorphism among psoriatic patients and controls from the same region [15]. Similar results for *FTO* rs9939609 and *FTO* rs1558902 were also confirmed in other studies involving psoriatic patients [16,17].

Our study provided inconclusive results for *FTO* rs9939609 concerning obesity among psoriatic patients. While the T allele was associated with a higher mean BMI among psoriatic patients compared to the control cohort, the A allele showed correlations with a higher mean WHR and disease severity indicators (PASI, BSA and DLQI). Tupikowska-Marzec et al. reported the A allele of *FTO* rs993960*9* to increase the risk of higher BMI, waist and hip circumference, a more severe course of psoriasis and elevated insulin concentration among psoriatic patients [16]. The PURE study in the general population of Polish origin found the A allele, particularly among male carriers, to be correlated with significantly higher mean body mass, BMI, WHR and waist and hip circumferences [55].

In our study, T allele carriers of *FTO* rs9939609 had greater SHAPS and PHQ-9 values, suggesting an increased risk of depressive disorder among psoriatic patients. However, in a systematic review, Zarza-Rebollo et al. emphasized that while there is strong evidence for a link between *FTO* rs9939609 and BMI, further research is needed to determine its role in depression–obesity comorbidity due to conflicting results [56].

Regarding *FTO* rs1558902, the GWAS analysis in the Polish population showed its potential role in the context of BMI, with the A allele being a risk factor for greater BMI among psoriatic patients [17]. However, our study did not confirm this association. Nevertheless, our findings indicated that the A allele of *FTO* rs1558902 could be a risk factor for an increased WHR ratio and more severe disease outcomes defined by BSA among psoriatic patients compared to the control group. Additionally, the T allele of *FTO* rs155890*2* might be a potential “protective allele” associated with less severe skin lesions due to lower BSA values. On the other hand, the T allele was associated with higher mean SHAPS values, suggesting a potential role in anhedonia. To date, no studies have evaluated this correlation.

## 5. Limitations of the Study

Our study possesses several limitations. Firstly, the results would benefit from confirmation in large-scale studies. Nonetheless, strict inclusive criteria applied in our study (including a BMI of ≥25 kg/m^2^, a minimum 6-month withdrawal period from systemic psoriatic treatment and the absence of other systemic concomitant diseases) contributed to minimizing bias. Secondly, we observed some disproportion between the sexes that met the inclusion criteria for the study, particularly in the psoriasis group. This further underscores the inclination of psoriatic men toward overweight and obesity. Certain clinical parameters were based on self-reported data provided by study subjects, such as the level of physical activity or dietary habits, introducing the possibility of bias. In future investigations, it would be valuable to expand the study, potentially including the pediatric population, allowing for early prophylaxis of cardiovascular events in this patient group. Additionally, we did not evaluate the long-term effects of psoriasis treatment methods on modifiable risk factors of obesity, such as body mass composition, which could offer an intriguing perspective for future studies.

## 6. Conclusions

Overweight and obesity among individuals with psoriasis require a holistic approach, considering various contributing factors. Adiposity stands out as a key and modifiable risk factor, influencing both psoriasis and its associated comorbidities, notably depression. Our study underscores the importance of prioritizing body composition evaluation over BMI, recognizing the limitations of BMI in defining complexities of body composition and its clinical implications. Additionally, two *FTO* gene polymorphisms, rs9939609 and rs1558902, are explored as potential genetic links between psoriasis and obesity, although further research is needed to validate these associations. These insights contribute to advancing our understanding of the multifaceted interplay between genetic, lifestyle and inflammatory factors in the context of psoriasis and obesity. 

## Figures and Tables

**Table 1:** Psoriasis patients’ group characteristics.

Variable	General *n* = 30	Men *n* = 26	Women *n* = 4	*p*-Value (Difference between Men and Women)
Mean age of onset of psoriasis [years ± SD]	26.80 ± 13.58	28.08 ± 13.62	18.50 ± 11.39	0.19
Type of psoriasis [%]				
Type I	24 [80.0%]	20 [83.33%]	4 [16.67%]	0.28
Type II	6 [20.0%]	6 [100.0%]	0 [0.0%]	
Family history of psoriasis [%]				
yes	19 [63.33%]	16 [84.21%]	3 [15.79%]	0.60
no	11 [36.66%]	10 [90.91%]	1 [9.09%]	
Nail psoriasis [%]				
presence	19 [63.33%]	16 [84.21%]	3 [15.79%]	0.60
absence	11 [36.66%]	10 [90.91%]	1 [9.09%]	
Psoriatic arthritis diagnosis [%]				
presence	5 [16.66%]	5 [100.0%]	0 [0.0%]	0.34
absence	25 [83.33%]	21 [84.0%]	4 [16.0%]	
Mean PASI [points ± SD]	18.43 ± 11.47	19.75 ± 11.71	9.88 ± 4.04	0.11
Mean BSA [% ± SD]	26.71 ± 23.05	29.20 ± 23.76	10.50 ± 5.26	0.06
Mean DLQI [points ± SD]	14.60 ± 8.41	14.38 ± 8.51	16.0 ± 8.75	0.75
Mean total cholesterol [mg/dL ± SD]	185.55 ± 27.54	186.12 ± 29.54	182.0 ± 9.06	0.79
Mean triglycerides [mg/dL ± SD]	128.03 ± 60.66	133.36 ± 63.04	94.75 ± 29.07	0.24
Mean HDL cholesterol [mg/dL ± SD]	44.86 ± 10.09	44.76 ± 10.38	45.67 ± 8.96	0.89
Mean LDL cholesterol [mg/dL ± SD]	117.07 ± 23.63	117.24 ± 23.99	115.67 ± 25.11	0.92

Abbreviations: SD—standard deviation, PASI—psoriasis area severity index, BSA—body surface area, DLQI—dermatological life quality index, HDL—high-density lipoprotein cholesterol, LDL—low-density lipoprotein cholesterol.

**Table 2:** Anthropometric measurements of the study group.

		Psoriasis Group				Control Group			
	
Variable		General *n* = 30	Men *n* = 26	Women *n* = 4	*p*-Value (Difference between Men and Women)	General *n* = 30	Men *n* = 20	Women *n* = 10	*p*-Value (Difference between Men and Women)
Mean BMI [kg/m^2^ ± SD]		32.03 ± 4.81	32.35 ± 4.95	29.92 ± 3.61	0.48	29.95 ± 7.14	28.88 ± 7.86	32.09 ± 5.12	0.17
Mean waist circumference [cm ± SD]		111.75 ± 13.19	113.54 ± 12.5	100.13 ± 13.14	0.056	102.47 ± 11.89	104.03 ± 12.02	99.35 ± 11.6	0.37
Mean hip circumference [cm ± SD]		113.35 ± 10.01	112.83 ± 10.41	116.75 ± 6.85	0.47	114.15 ± 11.0	112.7 ± 11.61	117.05 ± 9.55	0.2
Mean waist-to-hip ratio (WHR) [±SD]		0.99 ± 0.79	1.01 ± 0.09	0.86 ± 0.07	0.004	0.89 ± 0.07	0.92 ± 0.1	0.85 ± 0.05	0.007
Mean relative fat mass index (RFM) [% ± SD]		33.09 ± 4.95	31.68 ± 3.34	42.29 ± 3.76	0.003	33.4 ± 7.62	28.54 ± 4.05	42.48 ± 3.88	0.00002

Abbreviations: SD—standard deviation, BMI—body mass index, RFM – relative fat mass.

**Table 3:** Analysis of the total body water (TBW), extracellular body water (ECW) and ECW/TBW ratio in the study groups.

		Psoriasis Group				Control Group			
	
Variable		General *n* = 30	Men *n* = 26	Women *n* = 4	*p*-Value (Difference between Men and Women)	General *n* = 30	Men *n* = 20	Women *n* = 10	*p*-Value (Difference between Men and Women)
Total body water (TBW) [L ± SD]		51.31 ± 7.51	52.73 ± 6.32	38.97 ± 5.80	0.001	46.16 ± 8.52	50.74 ± 5.57	37.00 ± 5.34	0.00003
Total body water (TBW) [% ± SD]		49.05 ± 5.06	49.65 ± 4.99	43.83 ± 0.72	0.058	48.51 ± 5.75	51.50 ± 4.31	42.55 ± 2.87	0.00009
Extracellular body water (ECW) [L ± SD]		21.57 ± 2.99	22.05 ± 2.69	17.40 ± 2.52	0.024	19.69 ± 3.48	21.31 ± 2.85	16.45 ± 2.07	0.00009
Extracellular body water (ECW) [% ± SD]		21.36 ± 4.69	21.55 ± 4.91	19.60 ± 0.52	0.27	20.71 ± 2.13	21.56 ± 1.84	19.00 ± 1.61	0.003
Extracellular body water (ECW/TBW) [% ± SD]		42.12 ± 1.41	41.84 ± 1.17	44.57 ± 0.91	0.0006	42.85 ± 2.52	41.95 ± 2.39	44.66 ± 1.68	0.004

Abbreviations: SD—standard deviation.

**Table 4:** Analysis of the *FTO* gene rs9939609 polymorphism genotypes with clinical characteristics of overweight, obesity, depression and psoriasis disease severity in the study groups. *p*-Values in bold indicate statistical significance (*p* < 0.05).

		Psoriasis Group Genotype				Control Group Genotype					
	
Variable		Total *n* = 30	AA *n* = 17	AT *n* = 7	TT *n* = 6	*p*-Value (^1^)	Total *n* = 30	AA *n* = 15	AT *n* = 9	TT *n* = 6	*p*-Value (^1^)
Frequency [%]		100.0	56.67	23.33	20.0	0.83	100.0	50.0	30.0	20.0	0.83
Mean BMI [kg/m^2^ ± SD]		31.47 ± 4.10	32.51 ± 4.85	30.05 ± 2.79	30.17 ± 2.08	0.51	30.78 ± 5.15	31.10 ± 5.51	32.66 ± 5.17	27.17 ± 1.88	**0.01**
Mean waist circumference [cm ± SD]		104.32 ± 30.74	108.07 ± 30.52	109.50 ± 8.43	88.51 ± 43.13	0.81	102.57 ± 11.80	101.20 ± 13.65	105.44 ± 10.47	101.67 ± 9.48	0.45
Mean waist-to-hip ratio (WHR) [cm ± SD]		0.99 ± 0.10	1.02 ± 0.11	0.92 ± 0.09	0.98 ± 0.05	0.18	0.89 ± 0.07	0.87 ± 0.05	0.89 ± 0.07	0.96 ± 0.09	**0.03**
Mean relative fat mass index (RFM [% ± SD]		33.09 ± 4.95	32.39 ± 3.88	37.58 ± 6.46	29.86 ± 1.25	0.13	33.39 ± 7.62	34.21 ± 7.66	34.54 ± 9.27	29.63 ± 3.72	0.50
Mean fat mass (FM) [kg ± SD]		35.43 ± 12.32	37.83 ± 15.11	34.55 ± 6.25	29.49 ± 4.21	0.51	32.62 ± 11.82	33.81 ± 13.32	34.59 ± 12.38	26.70 ± 3.81	0.42
Mean fat mass (FM) [% ± SD]		33.06 ± 6.94	33.06 ± 7.87	36.40 ± 5.03	29.72 ± 4.45	0.52	33.95 ± 8.27	35.42 ± 7.11	34.77 ± 10.83	29.05 ± 5.56	0.18
Mean fat mass index (FMI) [kg/m^2^ ± SD]		10.88 ± 3.61	11.35 ± 4.35	11.47 ± 2.10	8.93 ± 1.46	0.51	10.76 ± 4.06	11.32 ± 3.88	11.68 ± 4.99	7.97 ± 1.43	**0.02**
Mean visceral adipose tissue (VAT) [L ± SD]		5.23 ± 2.29	5.97 ± 2.61	4.32 ± 1.68	4.07 ± 0.71	0.40	3.41 ± 1.86	3.27 ± 2.23	3.58 ± 1.59	3.50 ± 1.44	0.50
Mean phase angle (PhA) [φ ± SD]		5.81 ± 0.56	5.93 ± 0.53	5.28 ± 0.38	5.98 ± 0.56	**0.04**	5.64 ± 0.67	5.58 ± 0.60	5.76 ± 0.67	5.60 ± 0.91	0.28
Mean centile of phase angle (cPhA) [% ± SD]		51.31 ± 33.60	57.53 ± 32.04	30.67 ± 30.53	54.33 ± 38.12	0.71	54.40 ± 33.93	49.87 ± 32.21	61.22 ± 32.84	55.50 ± 43.58	0.31
Mean SHAPS [points ± SD]		1.67 ± 2.48	1.29 ±1.99	0.43 ± 0.53	4.17 ± 3.54	**0.006**	0.43 ± 0.89	0.40 ± 1.06	0.67 ± 0.87	0.17 ± 0.41	0.35
SHAPS >2 points [%]		6 [20.0%]	3 [17.65%]	0 [0.0%]	3 [50.0%]	0.07	1 [3.33%]	1 [6.67%]	0 [0.0%]	0 [0.0%]	0.60
Mean PHQ-9 [points ± SD]		8.93 ± 5.91	7.35 ± 4.79	8.14 ± 4.38	14.33 ± 7.84	0.51	5.17 ± 3.71	6.53 ± 4.27	4.22 ± 2.82	3.17 ± 2.04	0.31
PHQ-9 > 4 points [%]		23 [76.67%]	12 [70.59%]	5 [71.43%]	6 [100.0%]	0.32	16 [53.33%	11 [73.33%]	4 [44.44%]	1 [16.67%]	**0.05**
Mean PASI [points ± SD]		18.43 ± 11.47	19.16 ± 12.92	15.49 ± 8.17	19.82 ± 11.54	0.65					
PASI >10 points [%]		24 [80.0%]	14 [82.35%]	5 [71.43%]	5 [83.33%]	0.81					
Mean BSA [% ± SD]		26.71 ± 23.05	27.69 ± 23.48	25.21 ± 28.64	25.67 ± 18.08	0.90					
BSA > 10% [%]		26 [86.67%]	15 [88.24%]	6 [85.71%]	5 [83.33%]	0.95					
Mean DLQI [points ± SD]		14.60 ± 8.41	13.59 ± 9.25	16.14 ± 9.12	15.67 ± 5.32	0.51					
DLQI > 10 points [%]		17 [56.67%]	9 [52.94%]	4 [57.14%]	4 [66.67%]	0.84					
Mean total cholesterol [mg/dL ± SD]		185.56 ± 27.54	185.06 ± 30.18	182.71 ± 15.82	190.17 ± 34.32	0.13					
Mean triglycerides [mg/dL ± SD]		128.03 ± 60.66	119.63 ± 45.21	110.43 ± 50.81	171.00 ± 92.21	0.96					
Mean HDL cholesterol [mg/dL ± SD]		44.86 ± 10.09	46.44 ± 10.53	46.17 ± 11.70	39.33 ± 5.79	0.13					
Mean LDL cholesterol [mg/dL ± SD]		117.07 ± 23.63	118.69 ± 23.17	113.17 ± 20.25	116.67 ± 31.10	0.63					

(^1^) *p*-value—difference between mean values among the genotypes or difference between the frequency of genotypes in the study groups. Abbreviations: SD—standard deviation, BMI—body mass index.

**Table 5:** Analysis of the *FTO* gene rs1558902 polymorphism genotypes with clinical characteristics of overweight, obesity, depression and psoriasis disease severity in the study groups. *p*-Values in bold indicate the level of statistical significance (*p* < 0.05).

		Psoriasis Group Genotype				Control Group Genotype					
	
Variable		Total *n* = 30	AA *n* = 17	AT *n* = 7	TT *n* = 6	*p*-Value (^1^)	Total *n* = 30	AA *n* = 15	AT *n* = 9	TT *n* = 6	*p*-Value (^1^)
Frequency [%]		100.0	50.0	26.67	23.33	0.71	100.0	43.33	36.67	20.0	0.71
Mean BMI [kg/m^2^ ± SD]		31.47 ± 4.10	31.25 ± 4.39	31.59 ± 4.63	31.78 ± 3.29	0.49	30.78 ± 5.15	31.87 ± 6.24	30.25 ± 4.14	29.41 ± 4.47	0.48
Mean waist circumference [cm ± SD]		104.32 ± 30.74	104.42 ± 31.33	115.00 ± 11.63	93.44 ± 41.39	0.75	102.57 ± 11.80	105.46 ± 14.19	99.82 ± 9.61	101.33 ± 9.99	0.92
Mean waist-to-hip ratio (WHR) [cm ± SD]		0.99 ± 0.10	0.98 ± 0.08	1.02 ± 0.15	0.97 ± 0.07	0.09	0.89 ± 0.09	0.91 ± 0.06	0.87 ± 0.07	0.92 ± 0.09	0.92
Mean relative fat mass index (RFM) [% ± SD]		33.09 ± 4.95	32.45 ± 4.66	33.35 ± 3.20	34.18 ± 7.30	0.70	33.39 ± 7.62	35.37 ± 7.53	31.74 ± 7.96	32.15 ± 7.49	0.12
Mean fat mass (FM) [kg ± SD]		35.43 ± 12.32	34.76 ± 13.52	35.23 ± 13.20	37.05 ± 10.17	0.37	32.62 ± 11.82	34.36 ± 14.31	31.19 ± 10.01	31.49 ± 10.21	0.92
Mean fat mass (FM) [% ± SD]		33.06 ± 6.94	32.83 ± 7.45	30.96 ± 7.44	35.66 ± 5.19	0.37	33.95 ± 8.27	34.76 ± 7.73	33.55 ± 9.47	32.93 ± 8.39	0.48
Mean fat mass index (FMI) [kg/m^2^ ± SD]		10.88 ± 3.61	10.73 ± 3.97	10.31 ± 3.51	11.74 ± 3.22	0.37	10.776 ± 4.06	11.42 ± 4.20	10.42 ± 4.18	9.93 ± 4.01	0.10
Mean visceral adipose tissue (VAT) [L ± SD]		5.23 ± 2.29	5.51 ± 2.68	5.63 ± 2.31	4.26 ± 1.05	0.48	3.41 ± 1.86	3.82 ± 2.38	3.06 ± 1.47	3.15 ± 1.18	0.92
Mean phase angle (PhA) [φ ± SD]		5.81 ± 0.56	5.79 ± 0.47	5.83 ± 0.77	5.81 ± 0.62	0.55	5.64 ± 0.67	5.52 ± 0.69	5.76 ± 0.71	5.67 ± 0.59	0.27
Mean centile of phase angle (cPhA) [% ± SD]		51.31 ± 33.60	52.60 ± 31.82	45.71 ± 38.79	54.14 ± 36.78	0.85	54.40 ± 33.93	50.77 ± 39.94	55.18 ± 26.97	60.83 ± 36.17	0.49
Mean SHAPS [points ± SD]		1.67 ± 2.48	1.27 ± 2.12	0.87 ± 0.83	3.43 ± 3.69	0.31	0.43 ± 0.89	0.54 ± 1.19	0.36 ± 0.67	0.33 ± 0.52	0.89
SHAPS > 2 points [%]		6 [20.0%]	3 [20.0%]	0 [0.0%]	3 [42.86%]	0.12	1 [3.33%]	1 [7.69%]	0 [0.0%]	0 [0.0%]	0.51
Mean PHQ-9 [points ± SD]		8.93 ± 5.91	7.40 ± 4.42	10.13 ± 6.92	10.86 ± 7.43	0.51	5.17 ± 3.71	4.92 ± 3.15	5.55 ± 4.55	5.00 ± 3.79	0.63
PHQ-9 > 4 points [%]		23 [77.33%]	11 [73.33%]	7 [87.50%]	5 [71.43%]	0.69	16 [53.33%]	7 [53.85%]	7 [63.64%]	2 [33.33%]	0.49
Mean PASI [points ± SD]		18.43 ± 11.47	20.13 ± 9.91	22.16 ± 15.03	10.53 ± 6.71	0.39					
PASI > 10 points [%]		24 [80.0%]	14 [93.33%]	6 [75.00%]	4 [57.14%]	0.13					
Mean BSA [% ± SD]		26.71 ± 23.05	27.45 ± 17.89	39.25 ± 32.58	10.79 ± 8.71	0.09					
BSA > 10% [%]		26 [86.67%]	15 [100.0%]	7 [87.50%]	4 [57.14%]	**0.02**					
Mean DLQI [points ± SD]		14.60 ± 8.41	14.13 ± 8.85	16.50 ± 8.00	13.43 ± 8.77	0.54					
DLQI > 10 points [%]		17 [56.67%]	7 [46.67%]	7 [87.50%]	3 [42.86%]	0.12					
Mean total cholesterol [mg/dL ± SD]		185.56 ± 27.54	187.93 ± 28.33	189.14 ± 29.48	176.55 ± 26.09	0.70					
Mean triglycerides [mg/dL ± SD]		128.03 ± 60.66	121.20 ± 52.61	119.00 ± 47.75	151.71 ± 87.00	0.75					
Mean HDL cholesterol [mg/dL ± SD]		44.86 ± 10.09	49.29 ± 10.91	37.29 ± 6.60	43.57 ± 6.50	0.40					
Mean LDL cholesterol [mg/dL ± SD]		117.07 ± 23.63	117.64 ± 23.55	123.29 ± 27.45	109.71 ± 21.18	0.42					

(^1^) *p*-value—difference between mean values among the genotypes or difference between the frequency of genotypes in the study group. Abbreviations: SD—standard deviation, BMI—body mass index.

## Data Availability

Data are contained within the article and Supplementary Materials.

## Supplementary Materials

The following supporting information can be downloaded at: https://www.mdpi.com/article/10.3390/biomedicines12030517/s1, Figure S1: Definition criteria applied in the study; Table S1: Summary of other comorbid diseases among the study group. (a) Psoriasis patients. (b) Control group; Table S2: Custom designed oligonucleotide sequences for *FTO* rs1558902 for the tetra-primer amplification refractory mutation system PCR (T-ARMS-PCR) technique.
