# Supplementary material for: Analysis of Clinical and Genetic Factors of Obesity and Psoriasis Concomitance—The Influence of Body Mass Composition, Prevalence of Mood Disorders, Environmental Factors and FTO Gene Polymorphisms (rs9939609, rs1558902)
Source: Biomedicines. 2024 Feb 25;12(3):517. doi: 10.3390/biomedicines12030517 (PMC10968415; doi:10.3390/biomedicines12030517)
Supplement: Supplementary file 1 [file biomedicines-12-00517-s001.zip › Supplementary Materials.pdf]

Supplementary Materials:

Table S1. Definition criteria applied in the study.

**Parameter**	**Definiton Criteria**
Type of psoriasis	I - age of onset <40.0 years old
	II – age of onset 40.0 years old
PASI (Psoriasis Area Severity Index)	<10.0 points – mild
	10.0-15.0 points – moderate
	>15.0 points – severe
DLQI (Dermatological Life Quality Index)	0-1.0 points – no effect
	2.0-5.0 points - small effect
	6.0-10.0 points - moderate effect
	11.0-20.0 points – very large effect
	21.0-30.0 points - extremely large effect
SHAPS (Snaith-Hamilton Pleasure Scale)	> 2 points - clinically relevant anhedonia
PHQ-9 (Patient Health Questionnaire-9)	0-4.0 points - no effect
	5.0 - 9.0 points - mild depression
	10.0-20.0 points - moderate depression
	> 20.0 - severe depression
WHR (Waist-to-hip ratio)	≥ 0.85 - obesity in women
	≥ obesity in men
BMI (Body mass index)	<18.5 kg/m^2 - underweight
	18.5 - 24.9 kg/m^2 - normal weight
	25.0 – 29.9 kg/m^2 - overweight
	≥30.0 kg/m^2 - obesity

Table S2. Custom designed oligonucleotide sequences of *FTO* rs1558902 for the tetra-primer amplification refractory mutation system PCR (T-ARMS-PCR) technique.

**Polymorphism**	**Inner primer *forward* (*5′-3′*)**	**Inner primer *reverse* (*5′-3′*)**	**Outer primer *forward* (*5′-3′*)**	**Outer primer *reverse* (*3′-5′*)**
rs1558902	CTAGCCCTGTGGGTTTACATTTGA (A allele)	GTTGCAGCAATAACCTACCCAAA (T allele)	AGCAACTGCGATACAAGTGTTAGATATC	TCCCTAAATGAATACAGAGAGGAAAATG

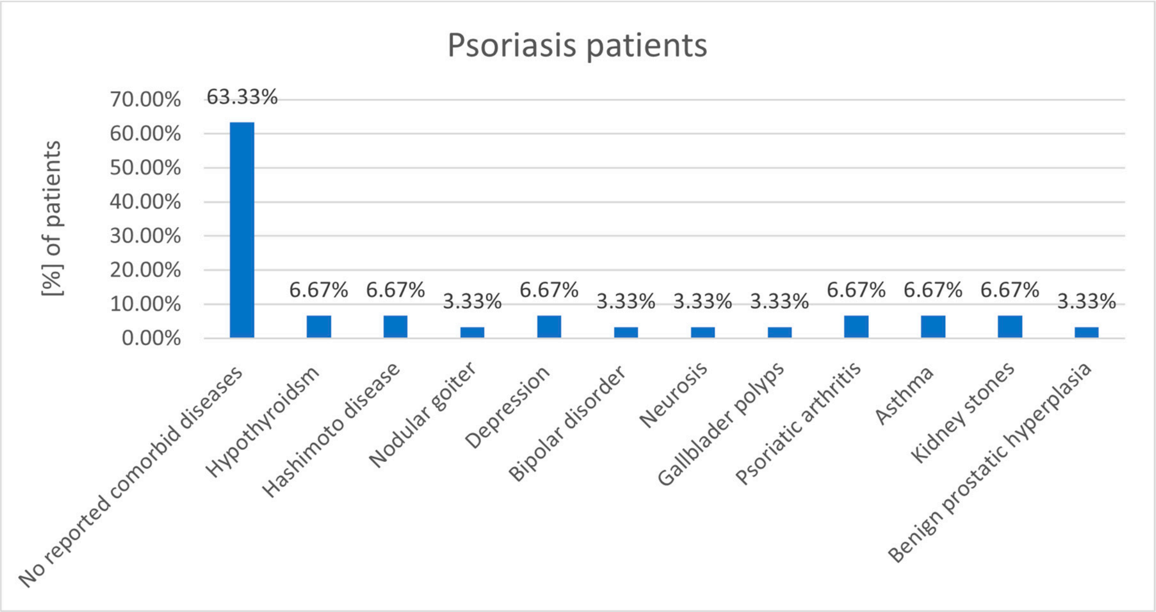

(**a**)

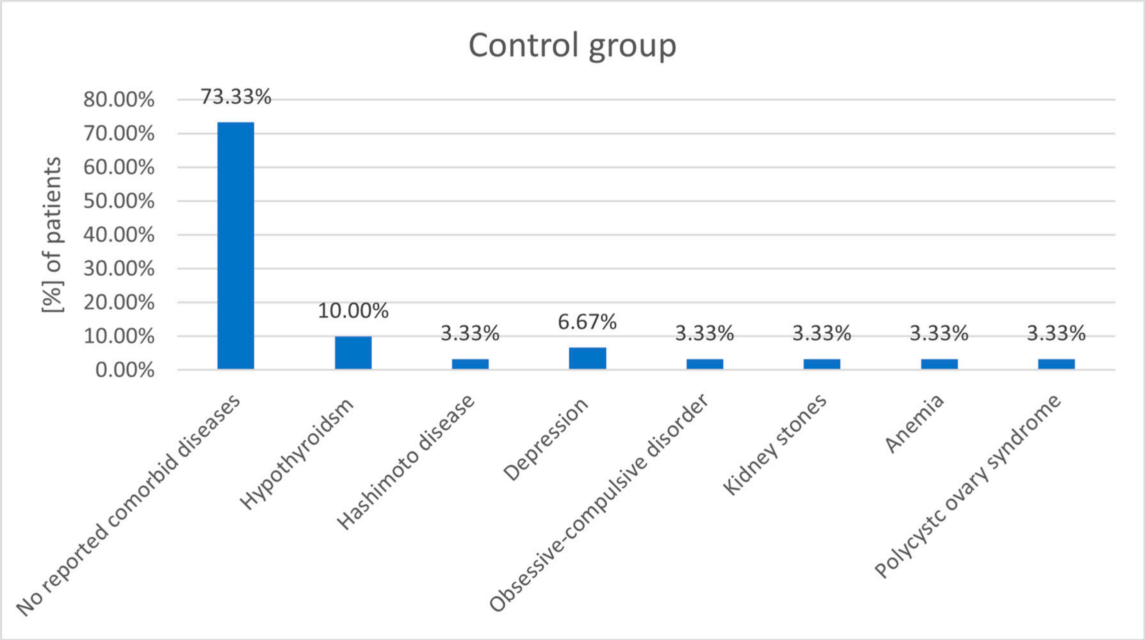

(**b**)

Figure S1. Summary of other comorbid diseases in the study groups. (**a**) Psoriasis patients. (**b**) Control group.
